# Mapping Evidence of Neonatal Resuscitation Training on the Practices of Unskilled Birth Attendants in Low-Resource Countries: Protocol for a Scoping Review

**DOI:** 10.2196/18935

**Published:** 2021-03-31

**Authors:** Adenike Adebola Olaniyi, Busisiwe Purity Ncama, Hafaza Amod

**Affiliations:** 1 School of Nursing and Public Health College of Health Sciences University of Kwazulu-Natal Durban South Africa

**Keywords:** neonatal resuscitation, newborn, neonatal, low- and middle-resource countries, training, birth, infant, baby, obstetrics, protocol, review

## Abstract

**Background:**

Competence in neonatal resuscitation of the newborn is very critical to ensure the safety and well-being of newborn infants. The acquisition of neonatal resuscitation skills by birth attendants improves self-efficacy, thereby reducing neonatal mortality as a result of asphyxia. Approximately one-quarter of all neonatal deaths globally are caused by birth asphyxia. The need for neonatal resuscitation is most imperative in resource-constrained settings, where access to intrapartum obstetric care is inadequate.

**Objective:**

This protocol describes the methodology of a scoping review on evidence of training in neonatal resuscitation and its association with practice in low-resource countries. The aim of the review is to map the available evidence of neonatal resuscitation training on the practices of unskilled birth attendants.

**Methods:**

The scoping review will use the Population, Concept, and Context (PCC) framework proposed by Arksey and O’Malley, refined by Levac et al, and published by Joanna Briggs Institute, while following the PRISMA-ScR (Preferred Reporting Items for Systematic Reviews and Meta-Analyses Extension for Scoping Reviews) guidelines. The search strategy was developed with the assistance of the college librarian. A number of databases of peer-reviewed research (PsycINFO and Wiley Online Library [via EBSCOhost], PubMed, MEDLINE with full text, Google Scholar [via ScienceDirect], and CINAHL Plus with full text [via EBSCOhost]) and databases committed to grey literature sources will be searched, and reference extraction will be performed. Two independent reviewers will screen and extract data, and discrepancies will be resolved by a third reviewer. The extracted data will undergo a descriptive analysis of contextual data and a quantitative analysis using appropriate statistical methods.

**Results:**

Data relating to neonatal resuscitation training and practices in low-resource settings will be extracted and included for analysis. We expect that the review will be completed 12 months from the publication of this protocol.

**Conclusions:**

This scoping review will focus on the review of evidence and provide an insight into the existing literature to guide further research and identify implementation strategies to improve the practices of unskilled birth attendants through the acquisition of skills and self-efficacy in neonatal resuscitation. The results of this review will be presented at relevant conferences related to newborn and child health and neonatal nursing studies and published in a peer-reviewed journal.

**International Registered Report Identifier (IRRID):**

DERR1-10.2196/18935

## Introduction

Annually, an estimated 10 million babies need help to initiate breathing, although all babies require an immediate assessment at birth [1]. Approximately, 5% to 10% of all babies born in health facilities need some measure of resuscitation, including tactile stimulation, airway clearance, and positioning [2]. According to the report of the World Health Organization (WHO), approximately 3% to 6% of neonates require basic neonatal resuscitation, which consists of simple initial steps as well as assisted ventilation [3]. Neonatal resuscitation refers to a set of interventions performed at the time of birth to support the establishment of breathing and circulation in newborns [4]. Most babies with primary apnea respond to stimulation, including drying and tactile stimulation, and will not require ventilation. Performing basic resuscitation with bag and mask is required for babies who cannot breathe and sufficient to resuscitate neonates with secondary apnea [4]. Advanced resuscitation (performed by skilled birth attendants), which comprises chest compressions, intubation, or medication, is essential for approximately 2% of all nonbreathing babies [5].

Identification of birth asphyxia in a newborn and prompt resuscitation requires immediate availability of a qualified individual, the appropriate equipment, and well-prepared action. Countries that wish to strengthen newborn resuscitation need to follow the suggested steps. In most low- and middle-income countries, birth attendants deliver more than 20 women a year. Therefore, in practice, health care institutions should introduce basic newborn resuscitation [3]. The call for neonatal resuscitation is most significant in low-resource settings, where access to intrapartum obstetric care is poor and the prevalence, mortality, and burden of long-time impairment from intrapartum-related events is highest. Delays in helping a nonbreathing neonate to establish ventilation, which occurs often in low-resource settings, may aggravate hypoxia and increase the need for assisted ventilation, thereby contributing to neonatal morbidity and mortality [1]. The impact of training birth attendants in neonatal resuscitation on mortality is restricted by the reduction of skills and knowledge over time and conveyance of skills into clinical practice [6]. Reducing neonatal death has been a rising challenge in low- and middle-resource countries in the past decade [7]. The development of low-cost interventions and their efficient delivery are desirable to reduce death from birth asphyxia. Increased mortality rates are somewhat ascribed to the shortage of trained birth attendants and a scarcity of resources [8]. Empowering unskilled birth attendants with adequate knowledge and skills in neonatal resuscitation can serve as an instrument of change for reducing newborn deaths [9]. The scoping review represents a suitable methodology for reviewing a large amount of research in order to generate an overview of the research undertaken on a topic and determine the range of studies that are available, summarize research results, and identify any evidence gaps [10]. In this context, our aim is to conduct a scoping review to examine the existing literature pertaining to the influence of training on practice. The review also seeks to map out available evidence of neonatal resuscitation training of unskilled birth attendants, examine the existing literature pertaining to practice among unskilled birth attendants, and identify gaps in the literature regarding future research surrounding neonatal resuscitation training on unskilled birth attendants’ practice where resources are limited.

## Methods

### Study Design

A scoping review will be conducted to identify and examine the existing research centered on the effects of neonatal resuscitation training on the practices of unskilled birth attendants in low-resource countries. In contrast to systematic reviews that aim to answer specific questions, scoping reviews produce a broad overview of the field. Hence, our study will be conducted using a methodological framework for scoping studies published by Arksey and O’Malley [8], which has been further developed by Levac et al [11] and the Joanna Briggs Institute [12]. As recommended by Tricco et al [13], this protocol will follow the relevant aspects of the PRISMA-ScR (Preferred Reporting Items for Systematic Reviews and Meta-Analyses Extension for Scoping Reviews) guidelines to ensure rigor and reduce bias in reporting the methodology [14]. Using this established protocol, we plan to review the existing literature systematically focusing on neonatal resuscitation training of unskilled birth attendants on practice and map out key concepts, thereby identifying the need for further research in this area. The framework includes the following stages: (1) identify the research question; (2) identify relevant studies; (3) perform study selection; (4) extract and chart the data; and (5) collate, summarize, and report the results.

### Stage 1: Identify the Research Question

The aim of this review is to identify what gaps exist within the research of neonatal resuscitation training of unskilled birth attendants and identify interventions that can be made to improve such gaps.

Furthermore, this study endeavors to create an understanding of how neonatal resuscitation training influences the practices of newborn care and what factors are essential in their implementation for achievement in a low-resource setting. We aim to provide answers to the following subquestions:

- What evidence is there that neonatal resuscitation training of unskilled birth attendants leads to competence in practice?
- What evidence is there that effective training improves newborn survival?
- What are the barriers and enablers to the efficient implementation of neonatal resuscitation?

This review will use the Population, Concept, and Context (PCC) framework (Table 1) recommended by Joanna Briggs Institute for scoping reviews [12] to determine the eligibility of the research questions. PCC is a more adaptable substitute for the Population, Intervention, Comparison, and Outcomes (PICO) framework for systematic reviews.

**Table 1:** Population, Concept, and Context framework for determination of the eligibility of the research questions for the review.

Criteria	Determinants
Population	The population for this review will be health care professionals who are unskilled. Skilled birth attendants—nurses, midwives, and doctors—are excluded from the review.
Concept/intervention	Neonatal resuscitation training
Context	While the literature citations for unskilled birth attendants in the African community are limited, we propose to extend the scope of this review to include low- and middle-resource countries and developing countries to increase the pool of studies included in this scoping review.

### Stage 2: Identify Relevant Studies

The research team developed a search strategy with the college librarian. Our literature search was open, including peer-reviewed literature and grey literature (ie, research not published in peer-reviewed journals). Using databases of peer-reviewed research (PsycINFO and Wiley Online Library [via EBSCOhost], PubMed, MEDLINE with full-text, Google Scholar [via ScienceDirect], and CINAHL Plus with full text [via EBSCOhost]), as well as the websites of the WHO and other organizations with policies and guidelines on neonatal resuscitation, a systematic search for relevant studies was conducted. The search was limited to articles published between January 2008 and January 2019, given that we wanted to examine the effects of neonatal resuscitation training on the practices of unskilled birth attendants over an 11-year period. We also performed a nonsystematic search (or grey literature search) of reports and guidelines from agencies (eg, WHO), using search engines designed to find evidence from published relevant interventions and studies in low- and middle-income countries, and selected grey literature reports from governmental and nongovernmental organizations.

The primary research teams were focused on using variations of the following Medical Subject Headings (MeSH) terms: resuscitation, practice, unskilled birth attendants, neonate/newborn, community health workers, training, and low/middle resource setting. In addition, a number of terms and keywords were searched, including “unskilled birth attendants,” neonatal resuscitation training, community health workers, low/middle resources setting, and unskilled birth attendants, along with the relevant subheadings. They were systematically combined into phrases using Boolean operators (AND, OR) to capture relevant fields. A complete list of the search terms is presented in Table 2. All researchers will update the number of publications identified and date of each literature search using Table 2. After searching, duplicates will be deleted, and the remaining papers will be exported to a web-based software platform that streamlines the inclusion eligibility screening for systematic reviews [15].

**Table 2:** Electronic search record.

Date searched	Keywords used^a^	Search engine or database used (number of publications)
12/4/2018	Neonatal resuscitation AND training AND practice AND community health workers AND birth asphyxia	EBSCOhost (9701); MEDLINE (543); PsycINFO (102)
15/4/2018	Neonatal resuscitation AND unskilled birth attendants AND low-resource setting	EBSCOhost (12,943); CINAHL Plus with Full Text (260); Education Resources Information Center (ERIC) (24)
17/4/2018	Neonatal resuscitation AND practice AND managing birth asphyxia in the community	Google Scholar (16,900)
20/4/2018	(“neonatal resuscitation” [MeSH terms] OR “neonatal resuscitation” [all fields]) AND “training” (MeSH terms) OR “community health workers” (all fields) OR “health workers” (all fields) AND “practice” (all fields)	PubMed (231); MEDLINE (876)
10/7/2018	Neonatal resuscitation AND practice of birth attendants AND perinatal birth asphyxia	EBSCOhost (46); Health Source: Nursing/Academic (5); Academic Search Complete (17)
12/1/2019	Neonatal resuscitation AND unskilled birth attendants	World Health Organization (41)

^a^“neonatal resuscitation” OR “newborn resuscitation training” OR “perinatal birth asphyxia” OR “birth asphyxia” OR “unskilled birth attendants” OR “community health workers” OR “managing birth asphyxia in the community” OR “practice” OR “low-resource setting.”

### Stage 3: Perform Study Selection

A library will be created for this review using EndNote X7.8 (Clarivate) referencing software. The investigators will search systematically and screen study titles from the database. All eligible study titles will be exported to the EndNote library. All duplicates will be removed, and abstract screening will be performed. Two reviewers (AAO and HA) will independently conduct abstract screening, followed by full-test screening of all studies selected using guidelines from the eligibility criteria. A third reviewer (BPN) will be consulted in cases of disagreement between the reviewers.

**Figure 1:**
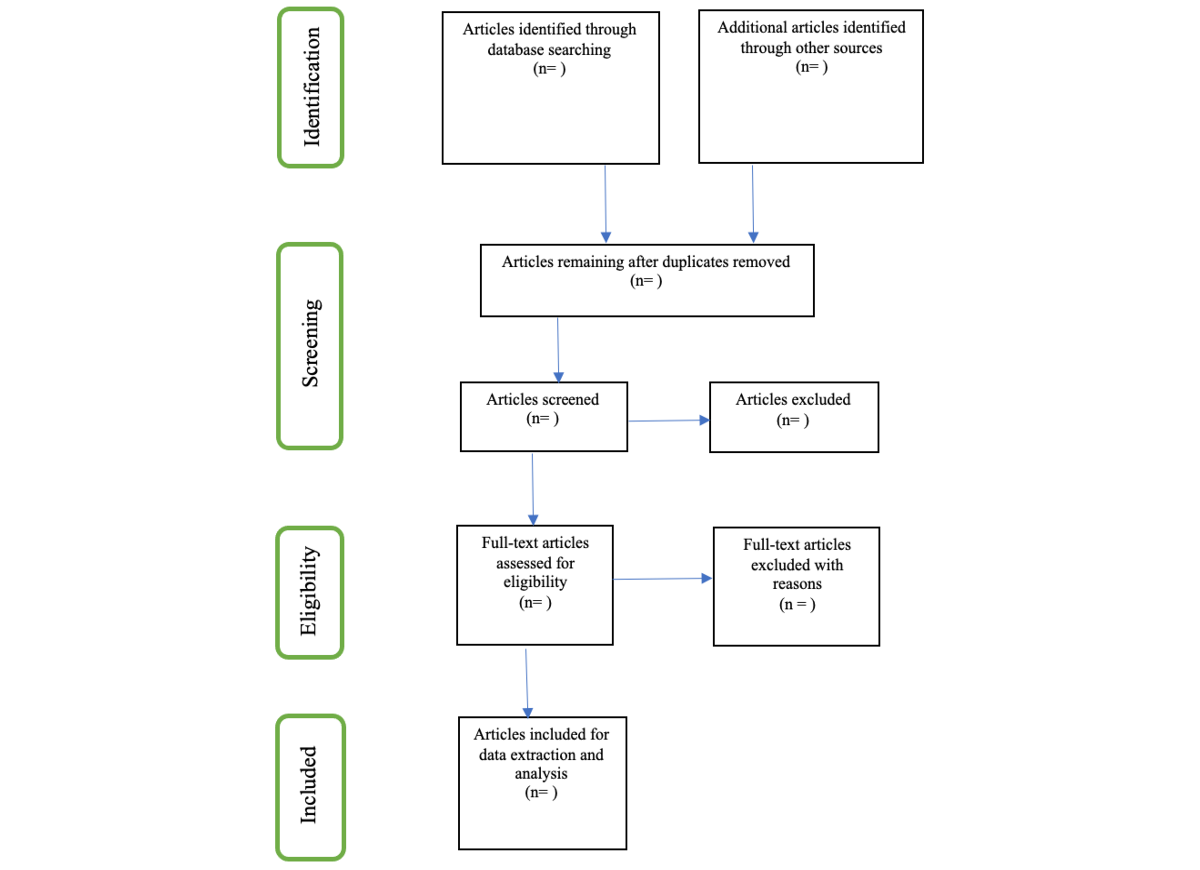
PRISMA-ScR (Preferred Reporting Items for Systematic Reviews and Meta-Analyses Extension for Scoping Reviews) flow diagram showing the phases of the literature search for extraction and selection of studies for the review [14].

Where articles are not available, authors will be contacted. We will exploit our local library services (University of KwaZulu-Natal) to retrieve articles to be included in the full article screening. Reporting will be done according to the PRISMA-ScR flow diagram [14] in Figure 1. Additional articles will be identified through reference mining of included studies. Subsequently, discussion will follow to establish a consensus on which papers will be included.

The inclusion criteria are as follows: (1) studies focused on neonatal resuscitation with unskilled birth attendants; (2) original research or reviews published in peer-reviewed journals relevant to the study; and (3) studies conducted in low- and middle-resource countries. Non–English-language studies will be excluded from the review. We will also exclude any studies without a focus on neonates or newborns and/or studies reporting preterm deaths.

### Stage 4: Extract and Chart the Data

Three independent team reviewers (AAO, HA, and BPN) will extract data from all eligible studies in triplicate using a standardized Google Forms tool for the abstracts. A data charting table will be used to extract and process the information from all studies. Data to be extracted and described will include the following: bibliographic details, study design, assessment of knowledge of health providers, practices, the effectiveness of training intervention, and study setting. Information specific to community health workers as unskilled birth attendants, descriptions of interventions as neonatal resuscitation training, and geographical locations of the studies will also be extracted. The team will autonomously design and compare the form for accuracy using the PCC framework of the review for the abstract [12]. Two reviewers (AAO and HA) will independently screen and select all references and process the relevant information from each included article. The third reviewer (BPN) will be consulted during the review to achieve harmony.

### Stage 5: Collate, Summarize, and Report the Results

The main aim of this study is to scope the existing evidence and summarize the findings as presented across articles. After data extraction is concluded, the research team will carry out a thematic analysis of the studies, where a narrative account of the data extracted from the included studies will be analyzed and an overview of the reviewed data will be provided. Data will be extracted and described according to the following features: bibliographic details, study design, assessment of knowledge of health providers, practices, effectiveness of training intervention, and study setting. Emerging themes from study results on neonatal resuscitation training for birth attendants who are unskilled will be coded by all authors independently. NVivo software (version 12; QSR International Pty Ltd [16]) will be expended to code the data from the included studies according to the above classifications. More importantly, the study team will scrutinize the meaning of findings as they relate to the overall aim of the study and discuss the implications for future research, practice, and policy.

## Results

We expect that the review will be completed 12 months from the publication of this protocol. The results will be reported based on the identified outcomes as specified above.

## Discussion

### Principal Findings

Basic neonatal resuscitation training can be successfully accomplished by health workers, resulting in a decline in intrapartum-related mortality [17]. Training programs in neonatal resuscitation can effectively increase the competence of health workers in conducting neonatal resuscitation and reduce potentially harmful practices [18]. Targeted research on neonatal resuscitation and its impact on the practice of community health workers who are unskilled birth attendants is needed. Training of community health care workers in neonatal resuscitation will enhance their skills and practice and improve the prevention of intrapartum-related deaths. Evidence from countries like India and Indonesia showed that community-based neonatal resuscitation training may be possible and helpful in reducing intrapartum-related mortality in settings with high rates of home births and delivery attendance by community health workers [19].

The proposed scoping review will generate findings that will aid in describing the links between neonatal resuscitation training and practices among community health workers who are unskilled birth attendants. This review will enable the authors to answer key questions, clarifying what is known and unknown about the links between this phenomenon in low- and middle-resource countries.

As such, the findings of this review will contribute to knowledge on this topic and impact skills for practice, policy, and research in the area of newborn survival and child health. Evidence generated in this scoping review may serve as a basis on which prevention strategies may be established for newborn mortality as a result of perinatal asphyxia.

Furthermore, this review will be significant in identifying research gaps and other ways in which reduction in newborn mortality can be achieved. Findings in this review will become relevant to researchers as evidence for the need for more work in this area.

### Strengths and Limitations of the Study

The protocol outlines a rigorous design that comprises an established research framework, a search strategy, and a selection process. The inclusion of grey literature and the use of peer-reviewed literature take into consideration a wide synopsis of various study designs and methodologies and emphasize the state of existing literature surrounding neonatal resuscitation training. The scoping review is an effective method for investigating and mapping comprehensive and different topics. The possible limitation regarding the amount of data for this scoping review study is that this study is not going to analyze the direct impact of training on mortality.

## Abbreviations

MeSH: Medical Subject Headings
PCC: Population, Concept, and Context
PICO: Population, Intervention, Comparison, and Outcomes
PRISMA-ScR: Preferred Reporting Items for Systematic Reviews and Meta-Analyses Extension for Scoping Reviews
WHO: World Health Organization
